# Pennogenin-3-*O*-α-*L*-Rhamnopyranosyl-(1→2)-[α-*L*-Rhamnopyranosyl-(1→3)]-β-*D*-Glucopyranoside (Spiroconazol A) Isolated from *Dioscorea bulbifera* L. var. *sativa* Induces Autophagic Cell Death by p38 MAPK Activation in NSCLC Cells

**DOI:** 10.3390/ph15070893

**Published:** 2022-07-19

**Authors:** Yo Sook Ki, Kyung-Sook Chung, Heon-Woo Lee, Jung-Hye Choi, Léon Azefack Tapondjou, Eungyeong Jang, Kyung-Tae Lee

**Affiliations:** 1 Department of Pharmaceutical Biochemistry, College of Pharmacy, Kyung Hee University, 26, Kyungheedae-ro, Seoul 02447, Korea; kiys018@gmail.com (Y.S.K.); adella76@hanmail.net (K.-S.C.); hwlee8972@gmail.com (H.-W.L.); 2 Oriental Pharmaceutical Science, College of Pharmacy, Kyung Hee University, 26, Kyungheedae-ro, Seoul 02447, Korea; jchoi@khu.ac.kr; 3 Department of Chemistry, Faculty of Science, University of Dschang, Dschang P.O. Box 183, Cameroon; tapondjou2001@yahoo.fr; 4 Department of Internal Medicine, College of Korean Medicine, Kyung Hee University, 26, Kyungheedae-ro, Seoul 02447, Korea; obliviona79@naver.com; 5 Department of Internal Medicine, Kyung Hee University Korean Medicine Hospital, 23, Kyungheedae-ro, Dongdaemun-gu, Seoul 02447, Korea

**Keywords:** spiroconazol A, non-small cell lung cancer (NSCLC), cell death, autophagy, p38 MAPK

## Abstract

In our previous study, we reported the isolation of pennogenin-3-*O*-α-*L*-rhamnopyranosyl-(1→2)-[α-*L*-rhamnopyranosyl-(1→3)]-β-*D*-glucopyranoside (spiroconazol A), a steroidal saponin, from the flowers of *Dioscorea bulbifera* L. var. *sativa*. In the present study, we aimed to investigate the effects of spiroconazol A on autophagy and its underlying mechanisms in A549 and NCI-H358 human non-small cell lung cancer (NSCLC) cells. Spiroconazol A inhibited the proliferation of NSCLC cells in a concentration- and time-dependent manner. To determine the type of programmed cell death induced by spiroconazol A, we performed a characterization of apoptosis in spiroconazol A-treated A549 cells. Our results showed that spiroconazol A significantly suppressed A549 cell viability but did not influence cell apoptosis because phosphatidylserine and caspase activation were not detected. Furthermore, spiroconazol A treatment upregulated the expression of LC3-II and autophagy-related Beclin-1 protein, suggesting that spiroconazol A induces autophagy in A549 cells. Moreover, spiroconazol A activated the phosphorylation of p38 mitogen-activated protein kinase (MAPK) but did not affect the phosphorylation of Janus kinase or ERK1/2. Notably, SB203580, a p38 MAPK inhibitor, had a significant inhibitory effect on spiroconazol A-induced autophagic cell death in A549 cells. Our results indicated that spiroconazol A-induced autophagy is dependent on p38 MAPK signaling and has potential as a therapeutic target in NSCLC.

## 1. Introduction

Lung cancer is a major cause of mortality worldwide, and non-small cell lung cancer (NSCLC) accounts for approximately 85% of all lung cancer cases [1]. Although surgery, radiation, and platinum-based chemotherapy, all of which induce apoptotic cell death, have proven the ability to improve survival rates in NSCLC, NSCLC is typically characterized by resistance to radiotherapy and chemotherapy and is often detected at a very late stage for surgical intervention. Additionally, Tungsukruthai et al. reported that conventional chemotherapy could not induce apoptotic cell death in 60% of NSCLC patients [2]. Hence, it is necessary to investigate novel compounds that induce apoptosis-independent cell death in patients who are not suitable for conventional chemotherapy.

Autophagic cell death, another cell death pathway, is considered an alternative therapeutic approach for cancer cells [3]. Autophagy (self-eating) is a tightly regulated lysosomal degradative process in which damaged cellular components, such as proteins and organelles, are engulfed and degraded in the lysosome to provide energy and perform biosynthesis of macromolecules. Autophagy is initiated by a response that confers protection against various environmental stresses, such as nutrient starvation, hypoxia, radiation, and chemotherapeutic agents, including arsenic trioxide, temozolomide, and fenretinide [4,5,6]. Although the mechanisms of autophagy in cancer are unclear, the induction of autophagic cell death is emerging as another possibility for cancer treatment.

Steroidal saponins isolated from medicinal herbs have been studied for their pharmacological effects, including antifungal, cytotoxic, and antitumor effects, owing to their structural diversity and important biological activities, such as cell cycle arrest, apoptosis, and autophagy [7,8,9]. In our previous study, both new and known steroidal saponins were isolated from the flowers of *Dioscorea bulbifera* L. var. *sativa* and the stem bark of *Dracaena mannii* Baker, and the cytotoxic activity of various isolated steroidal saponins against bladder carcinoma cells and antiedema activity was determined in a carrageenan-induced rat model [10,11]. Spiroconazol A showed moderate cytotoxicity against urinary bladder carcinoma cells (ECV-304 cells), and we prepared and compared the cytotoxicity of pennogenin and pennogenin glucosides including mannioside A (pennogenin-3-*O*-α-*L*-rhamnopyranosyl-(1->3)-β-*D*-glucopyranoside) and spiroconazol A (pennogenin 3-*O*-α-*L*-rhamnopyranosyl-(1->2)-[α-*L*-rhamnopyranosyl-(1->3)]-β-*D*-glucopyranoside). Based on the findings of our previous study on the structure–cytotoxicity relationship of hederagenin glycosides isolated from *Kalopanax pictus* Nakai, we acknowledge that the linkage of sugar moieties is very important for biological activities [11,12,13]. Of the three compounds (pennogenin, mannioside A, and spiroconazol A), spiroconazol A showed a potent cytotoxic effect against various cancer cells. However, the molecular mechanisms underlying the growth inhibitory efficacy of spiroconazol A in human NSCLC cells have yet to be elucidated. Therefore, as a part of our ongoing screening program to evaluate the anti-proliferative potentials of natural compounds, we investigated the effect of spiroconazol A on autophagic cell death and the underlying mechanism in human NSCLC A549 cells.

## 2. Results

### 2.1. Spiroconazol A Suppresses the Viability of Various Cancer Cells

With the isolated steroidal saponins (pennogenin, mannioside A, and spiroconazol A), as described in our previous study [10,11], we further investigated whether these compounds exerted a cytotoxic effect on various cancer cells using the 3-(4,5-dimethylthiazol-2-yl)-2,5-diphenyl-tetrazolium bromide (MTT) assay. Among the steroidal saponins, spiroconazol A (with a rhamnose attached to C-2 of *D*-glucose in mannioside A) had a potent cytotoxic effect (IC_50_ values of 2.07–10.95 μM for several tumor cell lines), while pennogenin did not exhibit any cytotoxic effect on various cancer cells at a dose of up to 100 μM (Table 1). Mannioside A (with a rhamnose attached to C-3 of *D*-glucose in pennogenin A) showed moderate cytotoxicity (IC_50_ values of 34.24–69.9 μM for several tumor cell lines). Because spiroconazol A exerted the maximum potent cytotoxic effect on human lung cancer cells (A549 IC_50_: 2.07 ± 0.18 μM and NCI-H358 IC_50_: 2.17 ± 0.08 μM) while spiroconazol A had lower cytotoxicity in the normal cell (MRC5 IC_50_: 6.43 ± 0.81 μM, Supplementary Figure S1), further experiments focused on unraveling the mechanism of spiroconazol A-induced cell death in human lung cancer cells.

### 2.2. Spiroconazol A Induces Apoptosis-Independent Cell Death

**Figure 1:**
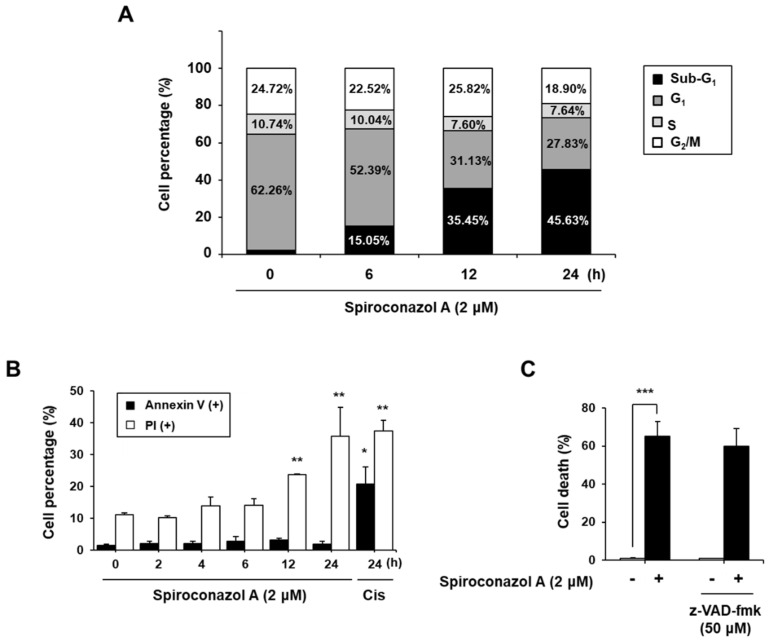
Non-apoptotic features were presented by spiroconazol A in A549 cells. (**A**) Cells were exposed to spiroconazol A (2 μM) and were stained with the PI solution. For detection of sub-G_1,_ indicating cell death, cells were determined by flow cytometry. (**B**) After treatment with spiroconazol A (2 μM) for the indicated times, cells were stained with FITC-conjugated Annexin V and PI (Cis: cisplatin) and then detected by flow cytometry. (**C**) After treatment with 50 μM z-VAD-fmk (broad caspase inhibitor) for 1 h, cells were treated with spiroconazol A (2 μM) for 24 h. Cells were stained with the PI solution and then examined by flow cytometry. Experiments were repeated at least three times, and data are expressed as mean ± S.D. * *p* < 0.05, ** *p* < 0.01, and *** *p* < 0.001 vs. control group by the Student’s *t*-test.

Because spiroconazol A inhibited cancer cell viability, we analyzed the effect of this saponin on cell death using propidium iodide (PI) by flow cytometry. Cells in the sub-G_1_ phase, which represented cell death, also increased in a time-dependent manner, but the cell cycle arrest was not affected in A549 cells (Figure 1A). To investigate whether spiroconazol A-induced cell death underwent apoptosis, we performed annexin V and PI double staining to examine the externalization of phosphatidylserine, which indicated apoptosis. Spiroconazol A did not increase the ratio of annexin V-positive cells relative to the control, whereas spiroconazol A induced a significant increase in the ratio of PI-positive cells in a time-dependent manner (Figure 1B). Furthermore, we confirmed that pretreatment with z-VAD-fmk, a broad-spectrum caspase inhibitor, did not block spiroconazol A-induced cell death. These results indicated that spiroconazol A-induced cell death occurred by inducing an apoptosis-independent pathway (Figure 1C).

### 2.3. Spiroconazol A Induces Autophagy in A549 and NCI-H358 Cells

**Figure 2:**
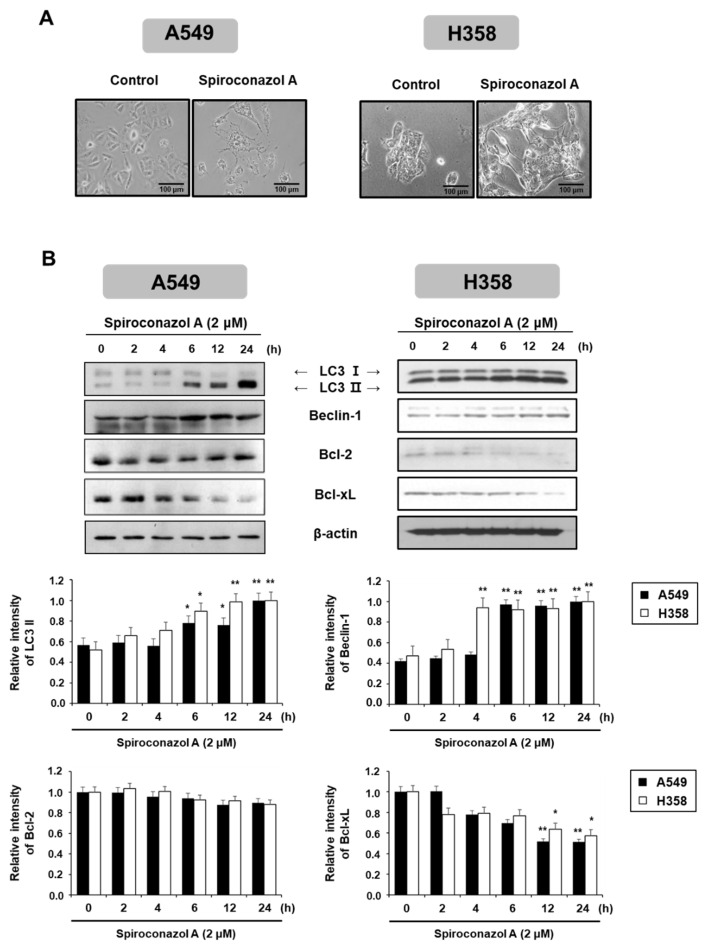
Autophagy induced by spiroconazol A in NSCLC cells. (**A**) Representative images showed the morphological changes in spiroconazol A (2 μM)-treated NSCLC cells using an OLYMPUS IX51 inverted microscope (Southend-on-Sea, Essex, UK). (**B**) Protein expression of cells exposed to 2 μM of spiroconazol A, using Western blot analysis. β-actin was utilized as an internal control. The relative optical density ratio was determined using a densitometric analysis program (Bio-Rad Quantity One^®^ Software, version 4.6.3 (Basic), Bio-Rad Laboratories Inc., CA, USA), normalized to the internal control. * p < 0.05, ** p < 0.01 vs. untreated A549 cells, by the Student’s *t*-test.

To better understand the caspase-independent cell death induced by spiroconazol A, we investigated other cellular responses related to cell death following spiroconazol A treatment. As shown in Figure 2A, compared with the control, spiroconazol A showed morphological features of cytoplasmic vacuole accumulation, unlike apoptotic bodies, and markedly decreased cell density. In particular, spiroconazol A treatment increased the size and number of cytoplasmic vacuoles compared with those in the control. Based on these morphological differences, we speculated that spiroconazol A-induced cytoplasmic vacuoles might be the products of autophagosome formation. In this regard, we detected autophagy-related protein expression using Western blot analysis. As shown in Figure 2B, treatment with 2 μM spiroconazol A markedly and time-dependently upregulated the protein expression levels of LC3-II and Beclin-1 in A549 and NCI-H358 cells. Previous studies reported that Bcl-2 family proteins interact with an evolutionarily conserved autophagy protein, Beclin-1, under normal conditions. However, under external conditions, the dissociation of Bcl-2 family proteins from Beclin-1 contributes to the induction of autophagy [14]. Our results showed that spiroconazol A (2 μM) treatment markedly reduced the expression levels of Bcl-xL, but not Bcl-2, in A549 and NCI-H358 cells (Figure 2B). These results suggest that Bcl-xL participates in the induction of autophagic cell death by spiroconazol A by dissociation from Beclin-1.

**Figure 3:**
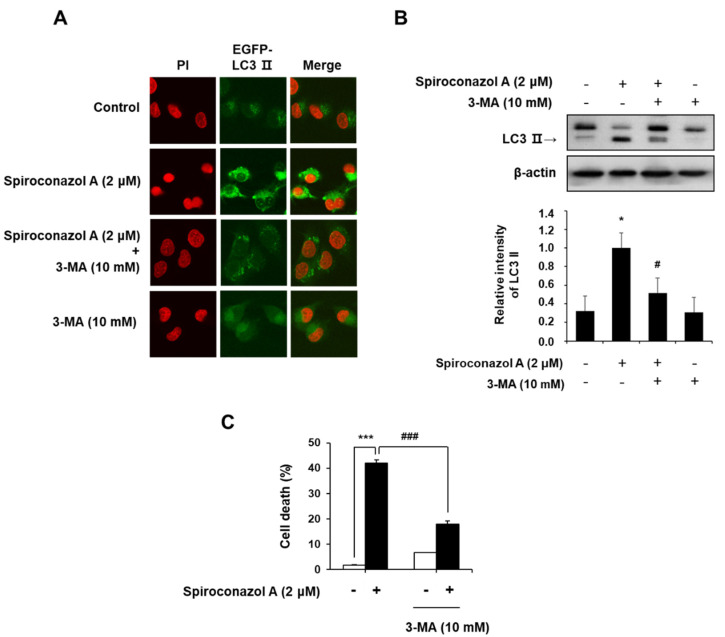
Cell death by spiroconazol A-induced autophagy. (**A**) The pEGFP-LC3-II-transfected A549 cells were treated with 2 μM of spiroconazol A for 24 h; immunofluorescence of pEGFP-LC3-II was detected by confocal fluorescence microscopy. After pretreatment with 10 mM of 3-MA for 1 h, cells were treated with 2 μM of spiroconazol A for 24 h and then examined by (**B**) Western blot analysis and (**C**) PI staining, respectively. Experiments were repeated at least three times, and data are expressed as mean ± S.D. The relative optical density ratio was determined using a densitometric analysis program (Bio-Rad Quantity One^®^ Software, version 4.6.3 (Basic), Bio-Rad Laboratories Inc., CA, USA), normalized to the internal control. * p < 0.05, *** p < 0.001 vs. untreated A549 cells and ^#^ *p* < 0.05, ^###^ *p* < 0.001 vs. spiroconazol A-treated A549 cells, by the Student’s *t*-test.

During autophagy, the degraded cytoplasmic contents are engulfed by double-membrane vesicles called autophagosomes, which can be observed on microscopy by the punctate formation [15]. To confirm spiroconazol A-induced autophagy, we monitored LC3-II localization in spiroconazol A-treated pEGFP-LC3-II-transfected cells. When pEGFP-LC3-II-transfected A549 cells were treated with spiroconazol A, immunofluorescence puncta of pEGFP-LC3-II were increased, indicating spiroconazol A-induced autophagy; however, pretreatment with 3-methyladenine (3-MA), an autophagy inhibitor, markedly attenuated spiroconazol A-induced LC3-II localization (Figure 3A). Western blot analysis revealed that spiroconazol A-induced LC3-II expression was blocked by 3-MA pretreatment (Figure 3B). We further investigated whether spiroconazol A-induced autophagy leads to either cell death or cell survival. Pretreatment with 3-MA markedly attenuated spiroconazol A-induced cell death in A549 lung cancer cells (Figure 3C), suggesting that spiroconazol A contributes to autophagic cell death in NSCLC cells.

### 2.4. The p38 MAPK Activation Plays an Important Role in Spiroconazol A-Induced Autophagic Cell Death in A549 Cells

**Figure 4:**
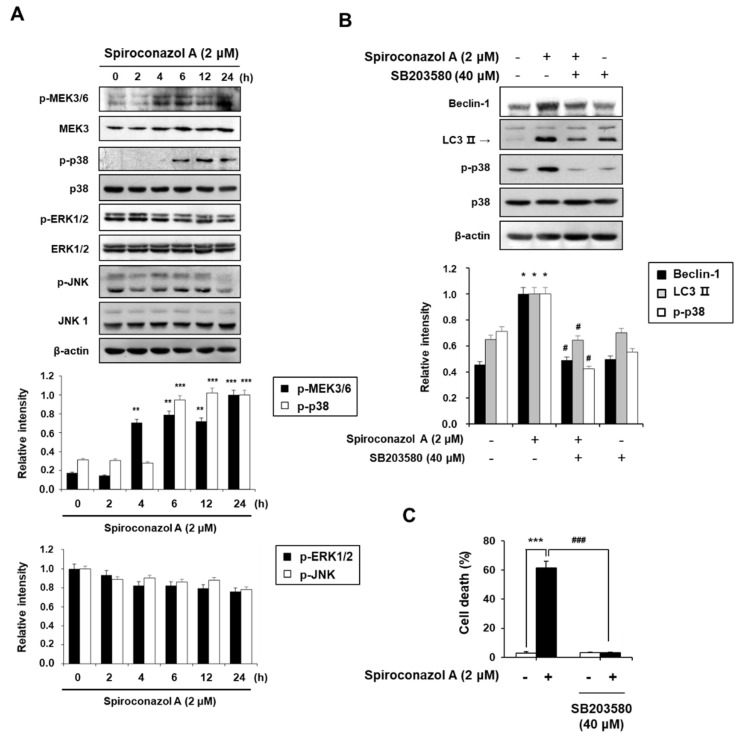
The p38 MAPK kinase signaling pathway is required in spiroconazol A-induced autophagic cell death. (**A**) After treatment with 2 μM of spiroconazol A for the indicated times, cells were examined by Western blot analysis. After pretreatment with 40 μM of SB203580 (p38 MAPK inhibitor), cells were treated with spiroconazol A for 24 h and then examined by (**B**) Western blot analysis and (**C**) PI staining, respectively. Experiments were repeated at least three times, and data are expressed as mean ± S.D. The relative optical density ratio was determined using a densitometric analysis program (Bio-Rad Quantity One^®^ Software, version 4.6.3 (Basic), Bio-Rad Laboratories Inc., CA, USA), normalized to the internal control. * p < 0.05, ** p < 0.01, *** p < 0.001 vs. untreated A549 cells and ^#^ *p* < 0.05, ^###^ *p* < 0.001 vs. spiroconazol A-treated A549 cells, by the Student’s *t*-test.

MAPKs play an essential role in eukaryotic cells by controlling cellular processes including cell proliferation, differentiation, migration, and programmed cell death such as autophagy [16]. In particular, p38 MAPK regulates autophagy in response to stress mediated by chemotherapeutic agents [17]. Therefore, to investigate whether MAPK signaling pathways are involved in spiroconazol A-induced autophagy, the activation of ERK1/2, JNK, and p38 MAPK was detected by Western blot analysis. As shown in Figure 4A, spiroconazol A treatment time-dependently and potently increased the phosphorylation of p38 MAPK and its upstream regulator p-MEK3/6, but it did not affect the phosphorylation of JNK or ERK1/2. Moreover, the levels of non-phosphorylated MEK3, p38 MAPK, JNK, and ERK1/2 were unaffected by spiroconazol A. To further confirm the role of p38 MAPK in spiroconazol A-induced autophagy, we exposed the cells to SB203580 (an inhibitor of p38 MAPK) before treatment with spiroconazol A. Our results demonstrated that SB203580 potently inhibited not only the upregulation of Beclin-1 and LC3-II protein expression (Figure 4B) but also autophagic cell death in spiroconazol A-treated cells (Figure 4C). These data strongly suggest that the p38 MAPK signaling pathway is a principal modulator of spiroconazol A-induced autophagy in NSCLC cells.

## 3. Discussion

The cytotoxicity of the 16 isolated steroidal saponins from the flowers of *D. bulbifera* L. var. *sativa* was determined by the MTT assay; spiroconazol A showed a potent cytotoxic effect (IC_50_ = 6.6 μM) on the urinary bladder carcinoma cell line ECV-304 [10]. However, cell death in various cancer cells and the underlying molecular mechanisms were not elucidated. In the present study, we described for the first time the autophagy-inducing activity of spiroconazol A and elucidated the underlying molecular mechanism.

Medicinal plants containing valuable substances with therapeutic effects play an essential role in the treatment of multiple diseases including cancer [18]. Most of the steroidal saponins containing a core structure of the hexacyclic ABCDEF ring system are known for their pharmacological effects, such as antitumor, anti-inflammatory, antiviral, antibacterial, and nerve sedation effects. Several studies have shown that steroidal saponin compounds have a wide range of antitumor activities, such as the inhibition of cell proliferation, induction of apoptosis and autophagy, and suppression of tumor invasion and metastasis [4,19,20]. In this study, we found that mannioside A (attachment of one sugar rhamnose to C-3 of *D*-glucose in pennogen-in A) had a significantly improved cytotoxic effect when compared with the pennigonin A. Furthermore, spiroconazol A contains an additional rhamnose moiety attached at site C-2 of *D*-glucose in mannioside A, and this moiety led to a markedly higher activity against various cancer cells (IC_50_ value from 10.95 to 2.07 μM) than those of mannioside A (IC_50_ value from 34.21 to 69.9 μM). An appropriate concentration of spiroconazol A could reduce the viability of NSCLC cells but did not influence apoptosis induction. Annexin V-positive cells and caspase activity, which are pivotal biomarkers of cancer cell apoptosis [21], were not affected by spiroconazol A treatment.

The retention of intracellular homeostasis is regulated by the balance between cell survival and death. In particular, autophagy maintains cell homeostasis, including cell survival (cytoprotective autophagy) [22,23] and cell death (autophagic cell death), depending on the cellular condition [24,25]. The key function of autophagy is the adaptation to metabolic stress through the removal of damaged proteins and organelles, which are deleterious to cell survival. Timosaponin AIII induces autophagy in AMPKα/mTOR-dependent and p53-independent pathways in hepatocellular carcinoma cells [26]. In addition, steroidal saponins such as spicatoside A and ophiopogonin B have been reported to suppress the growth of various cancer cells, which is attributed to the occurrence of autophagy through the inhibition of the PI3K/Akt signaling pathway [27,28]. Thus, in the present study, we observed the formation of a double membrane-bound vacuole, and the protein expression levels of LC3-II and Beclin-1 were enhanced in A549 and NCI-H358 cells after spiroconazol A treatment. We also demonstrated spiroconazol A-induced autophagy with the notable incorporation of GFP-LC3-II into autophagosomal membranes. The role of autophagy in tumors is complicated and involves several paradoxical functions [29]. Our data revealed that co-treatment with spiroconazol A and 3-MA (an autophagy inhibitor) markedly decreased spiroconazol A-induced LC3-II conversion, incorporation of GFP-LC3-II into autophagosomal membranes, and induction of cell death when compared with spiroconazol A treatment alone. Thus, spiroconazol A-induced autophagy is mainly caused by cell death rather than cell survival in A549 cells.

MAPK pathways, which include ERK1⁄2, JNK, and p38 MAPK, are involved in various biological processes such as cell differentiation, proliferation, and death [30]. The ERK1/2 cascade is part of the signaling pathways related to cell proliferation and survival [31], whereas the JNK and p38 MAPK pathways mediate stress signals and apoptosis [32]. The p38 MAPK also mediates crotoxin-induced autophagy of human lung carcinoma SK-MES-1 cells [33]. To elucidate the mechanism of autophagy, we investigated the role of MAPK in spiroconazol A-induced cell death. Because the MAPK pathway can mediate autophagy in ochratoxin A-treated cells [34], we examined the role of this signaling pathway in spiroconazol A-treated cells. In the present study, spiroconazol A was found to exclusively regulate p38 MAPK and its upstream MEK3/6 phosphorylation in A549 cells, supporting the idea that spiroconazol A-induced p38 MAPK activation affects autophagy signaling. SB203580 (a specific p38 MAPK inhibitor) markedly blocked spiroconazol A-induced Beclin-1 expression, LC3-II conversion, and cell death, all of which confirmed that the p38 MAPK pathway is mainly involved in spiroconazol A-induced autophagy in A549 cells.

In a recent study, oxidative stress and mitochondria were found to be targets not only for apoptosis but also for the autophagic pathway [35]. Intracellular reactive oxygen species (ROS) play a prominent role in carcinogenesis and the consequent cancer development [36]. Many studies have shown that excess ROS production suppresses the proliferation of lung cancer cells and induces apoptosis and autophagy [37,38,39]. The p38 MAPK signaling pathway plays an important role in cell survival in response to ROS stress [40]; 8 h after treatment with spiroconazol A, a considerable increase in ROS levels was observed by H_2_DCFDA staining (Supplementary Figure S2A). Moreover, *N*-acetylcysteine (NAC) suppressed spiroconazol A-induced cell death, indicating that NAC probably contributed to preventing spiroconazol A-induced autophagy by suppressing ROS production (Supplementary Figure S2B). In addition, the mitochondrial membrane potential was not changed in spiroconazol A-induced autophagy, indicating that MMP is not involved in autophagy induction (data not shown). Consequently, further mechanism studies are needed to investigate whether spiroconazol A affects autophagy through ROS-mediated p38 MAPK signaling in human NSCLC cells.

## 4. Materials and Methods

### 4.1. Materials

**Figure 5:**
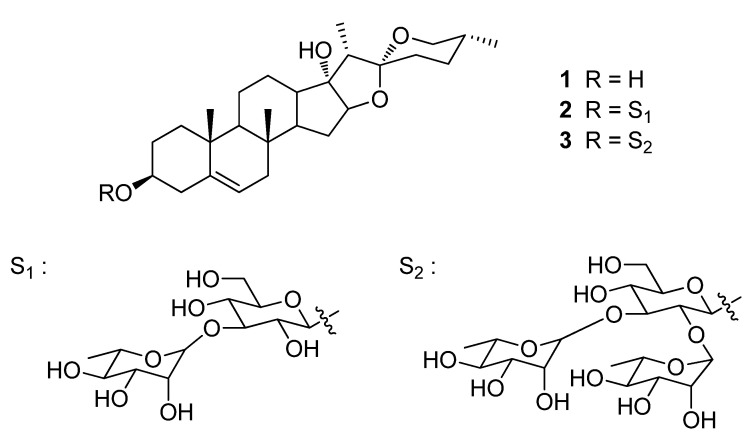
Chemical structures of pennogenin (**1**), mannioside A (**2**), and spiroconazol A (**3**) isolated from *D. bulbifera* L. var. *sativa*.

Pennogenin, mannioside A, and spiroconazol A (Figure 5) were obtained from Prof. Léon Azefack Tapondjou [10]. The purity of these compounds used in these studies was higher than 94% from the peak area in the HPLC chromatogram. This HPLC system consisted of a Waters 2695 Alliance system (Waters, Milford, MA, USA). Separations were achieved at 35 °C using a CAPCELL PAK C18 column (250 × 4.6 mm I.D., 5 µm, Shiseido, Tokyo, Japan). The mobile phase used for analysis consisted of a mixture of acetonitrile and 10 mM of sodium phosphate buffer (10:90, *v*/*v*). The mobile phase was adjusted to pH 5.7 with phosphoric acid and delivered at a rate of 0.5 mL/min. The column was eluted into a Waters 2996 photodiode array detector (PDA) (Waters, Milford, MA, USA) with the wavelength set at 210 nm. Data processing was performed with Empower Pro^®^ software (Waters, Milford, MA, USA). Fetal bovine serum (FBS), penicillin/streptomycin (PS), and RPMI 1640 medium in cell culture were obtained from Life Technologies Inc. (Chicago, IL, USA). The z-VAD-fmk was purchased from R&D Systems (Minneapolis, MN, USA) and Bradford solution was obtained from Bio-Rad Laboratories (Hercules, CA, USA). Antibodies for Bcl-2, Bcl-xL, β-actin, MEK3/6, p-ERK1/2, ERK1/2, p-JNK, JNK1, and p38 mitogen-activated protein kinase (MAPK) were purchased from Santa Cruz Biotechnology (Santa Cruz, CA, USA). LC3 I/II, p-MEK3/6, and p-p38 MAPK were purchased from Cell Signaling Technology (Danvers, MA, USA). A FITC (fluorescein isothiocyanate)-Annexin V Apoptosis Detection Kit I was purchased from BD Bioscience pharmingen (San Jose, CA, USA). MTT, 4′,6-diamidino-2-phenylindole (DAPI), PI, sodium dodecyl sulfate (SDS), dimethyl sulfoxide (DMSO), 3-MA, and other chemicals were purchased from Sigma Aldrich (St. Louis, MO, USA).

### 4.2. Cell Culture

Human cancer cells (A549, NCI-H358, HeLa, Caski, HT-29, HCT-116, ASPC-1, MiaPaCa-2, and MRC5) were obtained from the Korean Cell Line Bank (KCLB, Seoul, Korea) and were cultured according to the institution’s guideline.

### 4.3. MTT Assay

The IC_50_ of cytotoxicity was calculated with the results of the MTT assay, as previously described [41].

### 4.4. PI Staining Analysis

To evaluate the spiroconazol-induced cell death, PI staining analysis was used, as previously described [42].

### 4.5. Annexin V and PI Double Staining Assay

To identify apoptotic cells, spiroconazol A-treated cells were incubated with 100 μL of annexin V binding buffer containing PI and FITC-conjugated annexin V for 15 min in the dark. The cells were then analyzed using a FACS cytometer (Cytomics FC 500; Beckman Coulter, CA, USA).

### 4.6. Western Blot Analysis

After the extraction of cells [42], the extracted protein was quantified by Bradford protein assay. Proteins (30 μg) were separated by 8–15% SDS-PAGE gels via electrophoresis and transferred to poly-vinylidene difluoride (PVDF) membranes for immunoblotting. The membrane was exposed with primary antibodies in 5% skim milk in Tween 20/Tris-buffered saline (T/TBS) overnight at 4 °C. The primary antibody of the membrane was removed by T/TBS solution three times. Then, the immunoblotted membrane was exposed with horseradish peroxidase-conjugated secondary antibody for 2 h at 25 °C. Specific bands were visualized and developed by enhanced chemiluminescence using an ECL chemiluminescence substrate (Santa Cruz Biotechnology, CA, USA).

### 4.7. Construction of pEGFP-LC3-II and Observation with a Confocal Fluorescence Microscope

Human LC3-II cDNA was amplified from A549 cells by PCR with PrimeStar Taq DNA polymerase (Takara Bio, Shiga, Japan). The primers including the Kozak consensus sequence (GCC ACC) were constructed based on the previous literature [43]. The PCR primers for LC3-II were as follows: a forward primer with HindIII site; 5′-CCAAGCTTGCCACCATGCCGTCGGAGAAGAC-3′ and reverse primer with EcoRI site; and 5′-CCGAATTCTCACTTTTACACTGACAATTTC-3′. The cDNA of LC3-II was inserted into the pEGFP-VSVG mammalian expression vector (Addgene, Cambridge, MA, USA). After the transfection of pEGFP-VSVG and pEGFP-LC3 into cells using Lipofectamine 2000 (Invitrogen, Carlsbad, CA, USA), transfected cells were treated with spiroconazol A for 24 h. Then, cells were fixed with 4% paraformaldehyde for 5 min. After cell washing with PBS three times, cells were stained with 20 μL of PI (100 μg/mL) and observed by using a confocal fluorescence microscope (Carl Zeiss, Oberkochen, Germany).

### 4.8. Detection of ROS Generation

To measure spiroconazol A-induced intracellular ROS level, we used DCFH-DA, which is the most widely used fluorescent probe for the detection of intracellular oxidative stress. The spiroconazol A-treated cells were incubated with 20 μM of DCFH-DA for 30 min at 37 °C. The intracellular ROS level was measured by flow cytometry.

### 4.9. Statistical Analysis

Data are presented as the mean ± SD of triplicate experiments. Statistical analysis was performed using the Student’s *t*-test, and *p* values of < 0.05 were considered statistically significant.

## 5. Conclusions

In summary, spiroconazol A-induced cell death was mainly due to autophagy induction, which was associated with Beclin-1 and MAPK signaling. Therefore, we speculated that spiroconazol A may be a molecular-targeted therapeutic drug for the treatment of NSCLC. However, the mechanism underlying the relationship between p38 MAPK and ROS-related signaling responsible for autophagy induced by spiroconazol A remains uncertain. Hence, further studies are required to clarify the rational mechanism of spiroconazol A treatment in an animal model of NSCLC.

## Figures and Tables

**Table 1:** Cytotoxic effect of the steroidal saponins on various cancer cells.

Cell Line	Origin	IC_50_ (μM) ^(a)^		
		Pennogenin	Mannioside A	Spiroconazol A
A549	Human lung adenocarcinoma	<100	45.77 ± 7.59	2.07 ± 0.18
NCI-H358	Human bronchioalveolar carcinoma	<100	52.39 ± 5.30	2.17 ± 0.08
HeLa	Human cervix adenocarcinoma	<100	69.09 ± 4.92	8.21 ± 2.46
Caski	Human cervix epidermoid carcinoma	<100	54.91 ± 12.92	10.95 ± 0.62
HT-29	Human colorectal adenocarcinoma	<100	34.24 ± 2.73	4.94 ± 0.73
HCT-116	Human colorectal carcinoma	<100	68.33 ± 3.94	2.55 ± 0.05
ASPC-1	Human pancreatic adenocarcinoma	<100	55.59 ± 7.85	3.54 ± 0.75
MiaPaCa-2	Pancreatic ductal adenocarcinoma	<100	60.09 ± 7.77	2.18 ± 0.16

^(a)^ IC_50_ is the concentration of the steroidal saponins that results in a 50% reduction in cell viability relative to the control. Data are expressed as mean ± S.D.

## Data Availability

Data is contained within the article and Supplementary Material.

## Supplementary Materials

The following supporting information can be downloaded at https://www.mdpi.com/article/10.3390/ph15070893/s1. Figure S1: The cytotoxic effect of spiroconazol A on normal (MRC5 human fetal lung fibroblast) and lung cancer cells (A549 and H358 cells); Figure S2: Effect of spiroconazol A on intracellular ROS generation in A549 cells.
