# Supplementary material for: Pennogenin-3-O-α-L-Rhamnopyranosyl-(1→2)-[α-L-Rhamnopyranosyl-(1→3)]-β-D-Glucopyranoside (Spiroconazol A) Isolated from Dioscorea bulbifera L. var. sativa Induces Autophagic Cell Death by p38 MAPK Activation in NSCLC Cells
Source: Pharmaceuticals (Basel). 2022 Jul 19;15(7):893. doi: 10.3390/ph15070893 (PMC9319756; doi:10.3390/ph15070893)
Supplement: Supplementary file 1 [file pharmaceuticals-15-00893-s001.zip › pharmaceuticals-1788513-supplementary.pdf]

Supplementary Figures

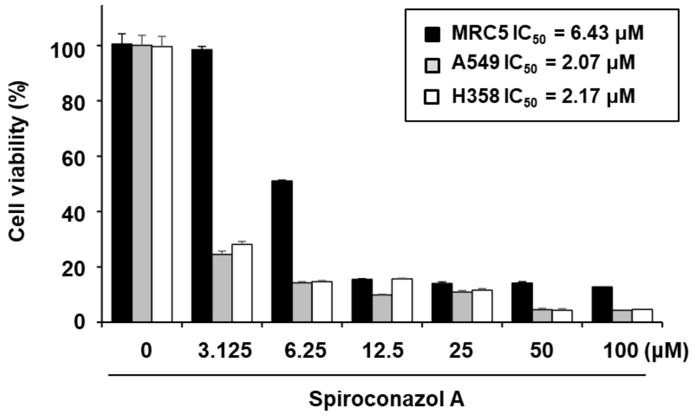

Supplementary Figure S1. The cytotoxic effect of spiroconazol A on normal (MRC5 human fetal lung fibroblast) and lung cancer cells (A549 and H358 cells). Cells were treated with various concentrations of spiroconazol A for 48 h. The cell viability was determined by MTT assay.

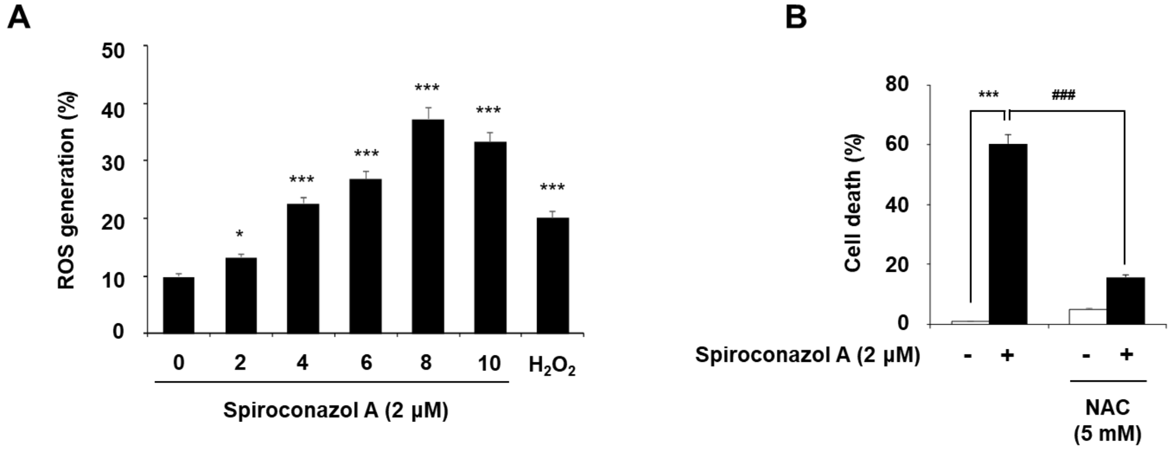

Supplementary Figure S2. Effect of spiroconazol A on intracellular ROS generation in A549 cells. (A) Cells were treated with spiroconazol A or H_2O_2 for the indicated time. ROS levels were determined with a DCFH-DA staining and the fluorescent intensity was measured by flow cytometry. (B) The effect of *N*-acetylcysteine (NAC) on cell death was examined in spiroconazol A-treated A549 cells. $^{*}p < 0.05$, $^{***}p < 0.001$ vs. untreated A549 cells and $^{###}p < 0.001$ vs. spiroconazol A-treated A549 cells, by the student's *t*-test.
